# Maternal immunization with pneumococcal surface protein A provides the immune memories of offspring against pneumococcal infection

**DOI:** 10.3389/fcimb.2023.1059603

**Published:** 2023-03-23

**Authors:** Masamitsu Kono, Takuro Iyo, Daichi Murakami, Hideki Sakatani, Denisa Nanushaj, Muneki Hotomi

**Affiliations:** ^1^ Department of Otorhinolaryngology-Head and Neck Surgery, Wakayama Medical University, Wakayama, Japan; ^2^ Department of Otorhinolaryngology-Head and Neck Surgery, Kinan Hospital, Tanabe, Japan

**Keywords:** maternal immunization, *Streptococcus pneumoniae*, PspA, immunological memory, invasive infection

## Abstract

**Introduction:**

*Streptococcus pneumoniae* (*S. pneumoniae*) is one of the most widespread pathogens in the world and one of the largest infectious causes of infant mortality. Although current vaccines have various benefits, antibiotic resistance and the inability to vaccinate infants less than one year old demands the development of new protective strategies. One strategy, ‘maternal immunization’, is to protect infants by passive immunity from an immunized mother, although its mechanism is still not fully understood.

**Materials and methods:**

The current study aimed to acquire immunity against *S. pneumoniae* in infants by maternal immunization with pneumococcal common antigen, pneumococcal surface protein A (PspA). Four-week-old female mice were immunized with recombinant PspA intranasally twice a week for three weeks. Females were mated with age-matched males after immunization, and delivered offspring.

**Results:**

The week-old offspring derived from and fostered by immunized mothers had more anti-PspA-specific antibody producing cells in the spleen than those derived from sham-immunized mothers. The offspring were raised up to four weeks old and were subcutaneously stimulated with recombinant PspA. The levels of anti-PspA IgG in sera after stimulation were significantly higher in the offspring derived from the immunized mothers and the induced specific antibody to PspA showed protective efficacy against systemic pneumococcal infection.

**Discussion:**

Maternal immunization is suggested to be able to provide a sustained immune memory to offspring. The current study would be a milestone in the field of maternal immunization toward a universal pneumococcal vaccine.

## Introduction

*Streptococcus pneumoniae* (*S. pneumoniae*) is an asymptomatic colonizer of the nasopharynx, but can cause severe invasive infectious diseases, such as meningitis, bacteremia and pneumonia. The risk of pneumococcal diseases is the highest in the neonatal period, especially in developing countries (Weiser et al., 2018). Babies are at particular risk because of their immature immune system, and neonatal natural killer cells are less responsive toward interleukin (IL)-2 and IL-15 and therefore produce lower interferon (IFN)-γ (Simon et al., 2015). Monocytes and macrophages are not fully mature in babies and preterm children (Kollmann et al., 2017), and studies have shown that newborn’s serum complements are lower than in adults, meaning lower host defense, opsonization level and antibody production (Yu et al., 2018). Furthermore, babies’ inability to make a neutrophil extracellular trap predisposes them to inefficiency against the bacteria (Lipp et al., 2017).

Vaccine development remains crucial in increasing infant protection against pathogens. Current pneumococcal vaccines, such as pneumococcal polysaccharide vaccine 23, pneumococcal conjugate vaccine (PCV) 7 and PCV13, target polysaccharide capsules, one of the leading pneumococcal virulent factors. PCV is effective in adults and older children owing to enhancement of immunogenicity by conjugation of the carrier protein. In children under 2 years of age, however, PCV provides insufficient immune induction because of the inability to generate T cell-dependent immune responses to B cells (Selman et al., 2000). Because of a heterogeneity of immune maturity, there are a few infants who show impaired response to vaccines called as ‘non-responder’ (Ingels et al., 2018). Another concern regarding the current vaccines is selection of non-vaccine strains, serotype replacement; the most commonly-known example being the 13-valent pneumococcal conjugate vaccine in Europe (Reinert, 2004). Selection of common pneumococcal antigens is an urgent matter in development of novel universal vaccines.

Transferred maternal immunity would provide an ideal immunological situation for infants during their period of weakness before they reach immune maturation. The most widely researched and understood factor is passive immunity by transfer of antibodies from mother to baby (Marcotte and Hammarström, 2015). Maternal antibodies are transferred to babies in two ways. Immunoglobulin G (IgG), the most abundant placental secreted isotype, is transplacentally transferred to the fetus in the third trimester of pregnancy (Fouda et al., 2018). Colostrum is particularly rich in antibodies, mostly IgA (0.5–1.5 g/l) and it is more protective than the milk produced during the latter period of breastfeeding, which consists of oligosaccharides, antisecretory lectins and lysozyme, all important in host defense in the period where babies approach two years of age (Ballard and Morrow, 2013). Antibodies in the milk are ingested through the intestine by an active transportation system (Jakoi et al., 1985).

Maternal immunization is a promising concept that arose with growing understanding of the mechanisms of protection passed from mothers to the children. Animal studies have shown that immunizing adult females with pneumococcal surface protein A (PspA) can be effective in protection against pneumococcal infection in offspring (Briles et al., 1996; Katsurahara et al., 2008; Hotomi et al., 2011; Kono et al., 2011). PspA, a choline-binding protein, is one of the common antigens of *S. pneumoniae* which determines its virulence by inhibiting deposition of complement and opsonophagocytosis by neutrophils. More than 95% of pneumococcal strains are classified as Family 1 (PspA1) and Family 2 (PspA2) whose antibody shows high cross-reactivity each other (Lane et al., 2022). For these reasons, PspA has been recognized as one of the promising target antigens for a next-generation universal pneumococcal vaccine (Almudevar and Pichichero, 2017; Mukerji et al., 2018). These maternally transferred antibodies rapidly disappear after weaning, however, resulting in the susceptibility to pathogens during the period between weaning and immune maturation. Maternal factors, such as cytokines/chemokines and/or cells, appear to have a role in maturation of the immune system of infants (Molès et al., 2018; Fujimura et al., 2019; Han et al., 2020). Maternal immune factors might therefore affect the offspring’s immune status in the long term as well as during the nursing period. Here, we investigate active immune responses and sustained protection against pneumococcal disease in offspring derived from immunized mothers with pneumococcal common antigen PspA.

## Materials and methods

### Ethical statement

All animal studies were conducted in accordance with the ARRIVE guidelines (Animal Research: Reporting of *In Vivo* Experiments) and were approved by the institutional Animal Care and Use Committee (approval number: 551, 559).

### Bacterial strains

*S. pneumoniae* TIGR4, serotype 4 strain, was used in this study. TIGR4 is known as one of the virulent strains that can cause invasive pneumococcal infections (Ibrahim et al., 2004; Kerr et al., 2006; Chimalapati et al., 2011; Chan et al., 2019). The bacteria were grown at 37°C in Todd-Hewitt broth with 0.5% yeast extract (THY) until mid-log phase. The strain was stored in 20% glycerol at -80°C until use.

### Mice

Four-week-old BALB/cByJ mice were purchased from CLEA Japan, Inc. (Tokyo, Japan) and were maintained under specific pathogen-free conditions until an infection experiment.

### Production of recombinant PspA

**Figure 1:**
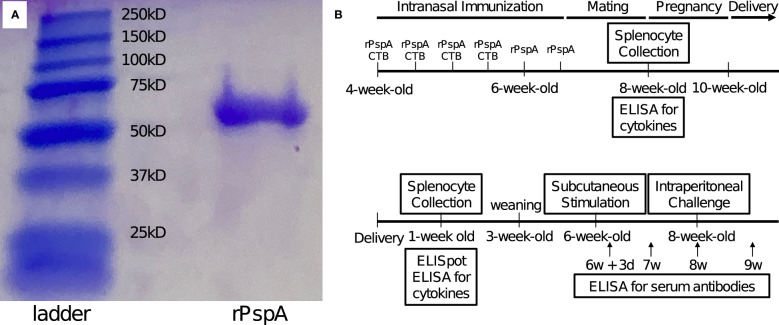
SDS-PAGE of rPspA and schematic of the experiment. **(A)** SDS-PAGE of rPspA after purification. Quantified rPspA was purified by nickel affinity chromatography. The appropriate size of the product and a single band were confirmed by SDS-PAGE. **(B)** Schedule from immunization of adult mice until experiments. Four-week-old female mice were immunized intranasally twice a week with rPspA 1 μg and CTB for the first two weeks and only rPspA for the last week. Control mice were given CTB only in the first two weeks and only saline during the last week. After immunization, the female mice were mated with male mice. Three weeks after mating, the offspring were obtained. Splenocytes of week-old offspring were analyzed for the ability to produce antibodies and cytokines. Subcutaneous stimulation of rPspA was performed at four weeks of age. The serum levels of anti-PspA antibodies were analyzed 3, 7, 14 and 21 days after the stimulation. At two weeks after the stimulation with rPspA (at six-week-old), offspring were intraperitoneally challenged with *S. pneumoniae*.

rPspA used for immunization was PspA2/TIGR4, which consisted of an α-helical region, as previously reported (Hollingshead et al., 2000; Kono et al., 2011). Its internal gene fragment of *pspA* was amplified by PCR from TIGR4. The amplified gene was sub-cloned by TOPO TA cloning kit (Invitrogen Inc., Carlsbad, CA, USA) and then cloned to the pET20b vector (Novagen Inc., Madison, WI, USA) by adjoining NcoI and XhoI sites. The pET20b vector containing *pspA* fragments was transformed into *E. coli* BL21 strain. To induce the expression, 1 mM isopropyl β-D-1-thiogalactopyranoside (IPTG) was used and then, the six-histidine-tagged rPspA was purified by nickel affinity chromatography. After quantification by protein assay kit (Bio-Rad), purified rPspA was adjusted to the desired concentration. The product size (about 67kD) and purity were confirmed by SDS-PAGE (**Figure 1A**).

### SDS-PAGE

rPspA was mixed 1:1 with a solution of 950 μl Laemmli Sample buffer (Bio-Rad, Hercules, CA, USA) + 50 μl β-mercaptoethanol (Fujifilm, Osaka, Japan), and boiled at 95°C for 5 minutes. Precision Plus Protein All Blue Standards (Bio-Rad) was used as a marker and electrophoresis was performed at 200 V, 3.0 A for 36 min until Sample buffer reached the bottom of the gel. After electrophoresis, bands were confirmed by Coomassie Brilliant Blue staining.

### Western blotting

Towbin Buffer was prepared by adding 5.76 g Tris base + 2.95 g Glycine + 200 ml methanol and up to 1 liter ddH_2_O. Extra thick blot paper (Bio-Rad) and nitrocellulose membrane (Amersham™ Protran™ NC 0.2, Cytiva, Tokyo, Japan) were used for electrophoresis, and each was hydrophilized with Towbin Buffer. Gels containing rPspA after SDS-PAGE were also equilibrated with Towbin Buffer for 15 min. The gels and membranes were sandwiched with blot paper and electrophoresed at 15V, 0.3A for 15 min using Trans-blot SD semi-dry transfer cell (Bio-Rad) and transferred to the membranes. The membrane was stained with Ponceau S solution to confirm that the protein was transcribed. The membrane was then blocked with PBS containing 1% BSA, then probed with 1:1,000 dilution of the pooled serum obtained from three mice immunized by rPspA and then probed with 1:100,000 dilution of streptavidin conjugated to horseradish peroxidase (Abcam plc, Cambridge, UK) for 1 hour each, at room temperature. Immunoreactive bands were visualized by chemiluminescence (ECL Prime Western Blotting Detection Reagent, Cytiva) and detected by imaging apparatus (LuminoGraph II, ATTO, Tokyo, Japan).

### Maternal immunization and experimental schedule

The outline of the experimental schedule is summarized in **Figure 1B**. Four-week-old female mice were pre-immunized intranasally with rPspA 1 μg twice a week for three weeks. During the first two weeks of immunization, mice received antigen with 4 µg of cholera toxin subunit B (CTB). To avoid any impact on pregnancy, only rPspA was applied to them twice in the last week. Control mice were given CTB only in the first two weeks and only saline during the last week. At the end of the immunization period, the female mice were mated with male mice for two weeks. Three weeks after mating, the offspring were obtained. To confirm that the mice immunized with rPspA produced antibodies against PspA, the pooled serum of three immunized mice were analyzed by western blotting at the age of 8-week-old (**Supplementary Figure**).

At the age of a week, splenocytes were collected for evaluation of abilities to produce anti-PspA antibodies and cytokines. Three-week-old offspring were separated from mother mice. At six weeks old, the offspring were subcutaneously stimulated with a single dose of 10 µg of rPspA without any adjuvants under a light anesthesia by isoflurane. The blood samples were obtained for evaluating serum antibodies against PspA at day 3, day 7, day 14, and day 21 after the stimulation with rPspA.

### Division of offspring

The offspring was divided into 4 groups as follows in order to evaluate the impact of feeding status (**Table 1**). In Group A, offspring derived from PspA-immunized mother were breast-fed by their PspA-immunized mother. In Group B, offspring derived from sham-immunized mother were breast-fed by PspA-immunized mother. In Group C, offspring derived from PspA-immunized mother were breast-fed by sham-immunized mother. In Group D, offspring derived from sham-immunized mother were breast-fed by sham-immunized mother.

**Table 1:** Four groups of the offspring.

	Group A	Group B	Group C	Group D
birth mother	immunized	sham-immunized	immunized	sham-immunized
foster mother	immunized	immunized	sham-immunized	sham-immunized

### Preparation of splenocytes

Murine spleens were removed after euthanization and placed in petri dishes with 5 ml PBS. The spleens were placed between two glass slides and crushed. A syringe was used to collect the spleen remnants in the petri dish and they were transferred in a tube, which was centrifuged at 300 g for 5 min. The supernatant was discarded and the pellet was resuspended in 1 ml red blood cell lysis buffer and incubated for 1 min. The cells were washed with PBS and isolated by using a 70-μm cell strainer into ice cold PBS containing 2% fetal bovine serum.

### Enzyme-linked immunospot assay

The procedure of ELISpot assay was performed according to the manufacturer’s protocol (KPL, Inc. Gaithersburg, MD, USA). The splenocytes (100,000 cells/well) of the offspring were incubated with 5 µg of rPspA overnight at 37 °C with 5% of CO_2_. The cells were then incubated in polyvinylidene difluoride membrane 96 wells coated with rPspA (10 µg/ml) overnight at 37 °C with 5% of CO_2_. The splenocytes were removed by washing with PBS, the immobilized antibodies on the membrane were incubated with biotinylated detection antibody to mouse IgG (Southern Biotechnology Associates, Birmingham, AL, USA) for 1 hour, then incubated with alkaline phosphatase conjugates with streptavidin for 30 minutes at room temperature. The number of spots visualized by 5-bromo-4-chloro-3-indolyl-phosphate/nitro blue tetrazolium (BCIP/NBT) substrates were counted under a microscope (Olympus SZX16, Tokyo, Japan).

### Enzyme-linked immunosorbent assay for serum anti-PspA antibodies

At six weeks old, the offspring were subcutaneously stimulated with a single dose of 10 µg of rPspA without any adjuvants. Blood samples were collected after 3 days, 7 days, 14 days, and 21 days of the stimulation. Anti-PspA specific antibodies (IgM, IgG1, IgG2a, and IgG2b) in the sera of offspring were evaluated by ELISA, as previously reported (Hotomi et al., 2011; Kono et al., 2011). Ninety-six-well microplates (MaxiSorp, Nunc, Roskilde, Denmark) were coated with 50 μl of rPspA (2 μg/ml in PBS) overnight at 4 °C. After washing three times with PBS containing 0.05% Tween 20 (PBS-T), the wells were blocked for 1 hour with casein buffer (0.25% casein, 0.05% Tween 20 in PBS) at room temperature. Then, 50 μl of samples prediluted by 1/10 serially-dilluted by 1/3 with casein buffer were incubated at 4 °C overnight. After washing with PBS-T, the plate was incubated with 50 μl of biotinylated antibody (1:3,000) to mouse IgM, IgG1, IgG2a, or IgG2b (Southern Biotechnology Associates) diluted in casein buffer for 2 hours at room temperature. After washing with PBS-T, the plate was incubated with alkaline phosphatase-conjugated streptavidin (1:4,000) (Southern Biotechnology Associates) for 2 hours at room temperature. Color was developed with p-nitrophenyl phosphate (Sigma Chemical Co., St. Louis, MO, USA) and the optical density of each well was measured by a spectrophotometer at 405 nm. To determine a total amount of IgM and IgG subclasses, the wells were coated with anti-mouse IgM antibody, anti-mouse IgG1 antibody, anti-mouse IgG2a antibody, or anti-mouse IgG2b antibody (Southern Biotechnology Associates) instead of rPspA. As negative controls, we made wells without the sample, the secondary antibody, or alkaline phosphatase-conjugated streptavidin that were confirmed under the detection limit.

### Enzyme-linked immunosorbent assay (for cytokines

The splenocytes (100,000 cells/well) were incubated with 1 µg or 5 µg of rPspA overnight at 37 °C with 5% of CO_2_. For positive control, the cells were incubated with 1 µg of concanavalin A. For negative control, the cells were incubated with PBS. The supernatant was collected by centrifugation after the incubation. Quantitative evaluation of cytokines (IL-4, IFN-γ, and IL-17A) was performed using a dedicated ELISA kit, Quantikine (R&D systems, Inc. Minneapolis, MN, USA).

### Systemic pneumococcal infection model

At 8 weeks of age (2 weeks after the subcutaneous stimulation with rPspA), the offspring were inoculated intraperitoneally with pneumococcal cells at 1 x 10^2^ CFUs in 100 μl of sterile PBS under anesthesia. After the intraperitoneal infection, the offspring were monitored every 8 hours until they appeared severely sick, defined as the presence of all the following symptoms: decreased motor activity, shivering, messy hair. Sick mice were immediately humanely euthanized and the bacteremia was confirmed by blood culture of each mouse. Survival periods were expressed as times from inoculations of pneumococci to septicemia.

### Statistics

Comparisons between the two groups and more than two groups were evaluated by Mann Whitney’s U test and Kruskal-Wallis test respectively. Comparison of the survival after intraperitoneal challenge between the two groups was evaluated by Log rank test. Statistical differences were calculated by Prism 8 (Graphpad Software, La Jolla, CA, USA).

## Results

### The number of anti-PspA specific IgG producing cells were significantly higher in neonates derived from and breastfed by immunized mothers

**Figure 2:**
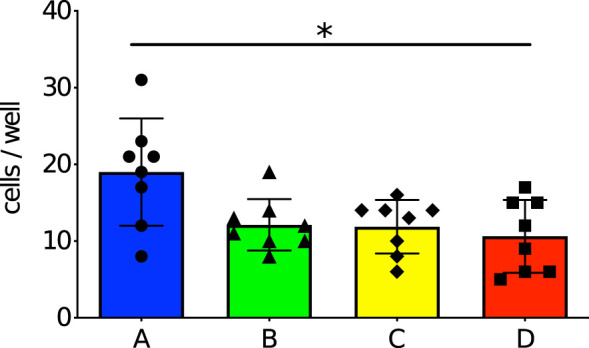
Number of anti-PspA specific IgG producing cells in the spleens of 7-day old offspring. The splenocytes were incubated with 5 µg of rPspA overnight, then incubated in polyvinylidene difluoride membrane 96 wells coated with rPspA overnight. After the cells were removed, the immobilized antibodies on the membrane were incubated with biotinylated detection antibody to mouse IgG, then incubated with alkaline phosphatase conjugates with streptavidin. The spots were visualized by BCIP/NBT. Circle and blue bar; Group A (n=8), triangle and green bar; Group B (n=8), diamond and yellow bar; Group C (n=8), square and red bar; Group D (n=8). Each dot represents individual data of the mouse. Each data is shown as mean with standard error of the mean. Kruskal-Wallis test with Dunn’s multiple comparison was performed for a comparison between the groups. *; *P*<0.05.

Ability of neonates’ splenocytes to produce anti-PspA specific antibodies was evaluated by ELISpot assay for 8 pups of each group. Seven-day-old offspring in Group A had a significantly greater number of anti-PspA specific IgG producing cells in the spleen than the offspring in Group D (**Figure 2**). This suggests that pups derived from and breastfed by immunized mothers were capable of producing anti-PspA specific antibodies, although they had no direct exposure to PspA. On the other hand, when the offspring were received only one of transplacental immunization and breastmilk from immunized mother, the increase of specific antibody producing cells was not observed.

### Specific immunological memory against PspA was observed among matured offspring derived from and breastfed by immunized mothers

**Figure 3:**
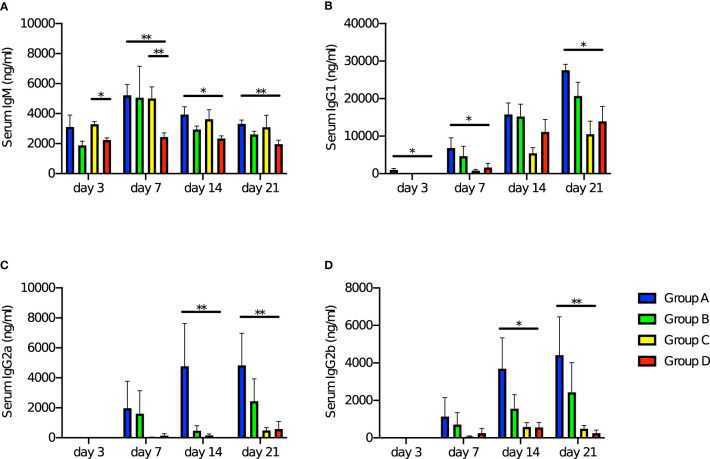
Change of levels of anti-PspA antibody in offspring’s sera with time after stimulation with rPspA. Four-week-old offspring were subcutaneously stimulated with a single dose of 10 µg of rPspA without any adjuvants. The levels of serum antibodies against PspA were evaluated by ELISA at day 3, day 7, day 14, and day 21 after the stimulation with rPspA. Data is shown as mean with standard error. Blue bar, green bar, yellow bar, and red bar represent Group A (n=9), Group B (n=8), Group C (n=9), and Group D (n=8) respectively. **(A)** serum IgM, **(B)** serum IgG1, **(C)** serum IgG2a, **(D)** serum IgG2b. Kruskal-Wallis test with Dunn’s multiple comparison was performed for comparison between the groups in each day of the blood sampling. *; *P*<0.05, **; *P*<0.01.

Investigation of the specific immunological memory by maternal immunization was performed by ELISA analysis of the blood collected on days 3, 7, 14 and 21 after the stimulation. We previously confirmed that maternal antibodies remarkably decreased within two weeks if the offspring were not breastfed by the immunized mother (Kono et al., 2011), or started decreasing at the age of three weeks even if they were breastfed by the immunized mother (Yamauchi et al., 2006), so this time the schedule was set to take samples at 6-weeks-old (more than two weeks after weaning). Anti-PspA specific IgM was positive among all groups on day 3 after PspA stimulation. The IgM antibody levels were highest on day 7 after the stimulation among offspring derived from immunized mother and/or nursed by immunized mother (Group A, B, and C). Especially in Group A, the IgM antibody titer was significantly higher than in Group D on day 7, day 14, and day 21 (**Figure 3A**). Anti-PspA specific IgG1 on day 3 after the stimulation was positive only among Group A. Although an increase in antibody titer was observed in all groups on day 7, day 14, and day 21, the antibody titer in Group A was significantly higher than that in Group D (**Figure 3B**). IgG2a and IgG2b showed similar change. The antibody could not be detected on day 3 after the stimulation in all groups, however an increase of the antibody titer was significantly higher in Group A on day 14 and day 21 after the stimulation than in Group D (**Figures 3C, D**).

### Production of IFN-γ and IL-17A by splenocytes was higher among immunized adult mice

**Figure 4:**
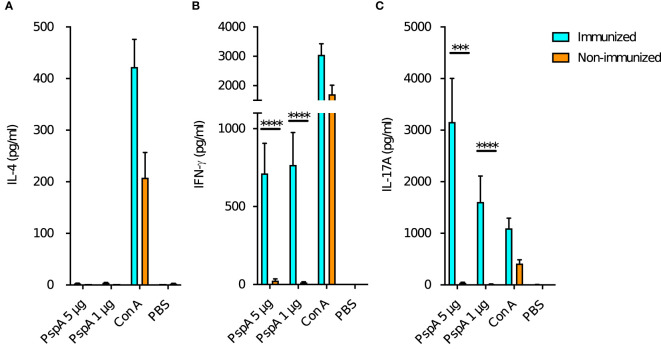
Cytokine production from maternal splenocytes by PspA stimulation. At the age of 8-week-old, the splenocytes from female mice were incubated with rPspA (1 or 5 µg) or concanavalin A (con A) for positive control. Cytokines in the supernatant were quantified by ELISA. **(A)** IL-4, **(B)** IFN-γ, **(C)** IL-17A. Data is shown as mean with standard error. Cyan bar and orange bar indicate immunized female (n=12) and non-immunized female (n=12) respectively. Mann-Whitney’s U test was performed for comparison between the groups. ***; *P*<0.001, ****; *P*<0.0001.

In order to evaluate changes in maternal immune status due to intranasal immunization, splenocytes of pre-pregnant adult female mice were stimulated with rPspA and production of cytokines (IL-4, IFN-γ, and IL-17A) was measured for the immunized (n=12) and non-immunized female (n=12). The production of IL-4 was not observed neither in the immunized or non-immunized mice (**Figure 4A**). The splenocytes of the immunized mice significantly produced IFN-γ after the stimulation with of rPspA (**Figure 4B**). On the other hand, IL-17A production was significantly higher in the immunized female and dose-dependently increased by rPspA stimulation (**Figure 4C**).

### Production of IFN-γ and IL-17A by splenocytes was higher among neonates derived from and breastfed by immunized mothers

**Figure 5:**
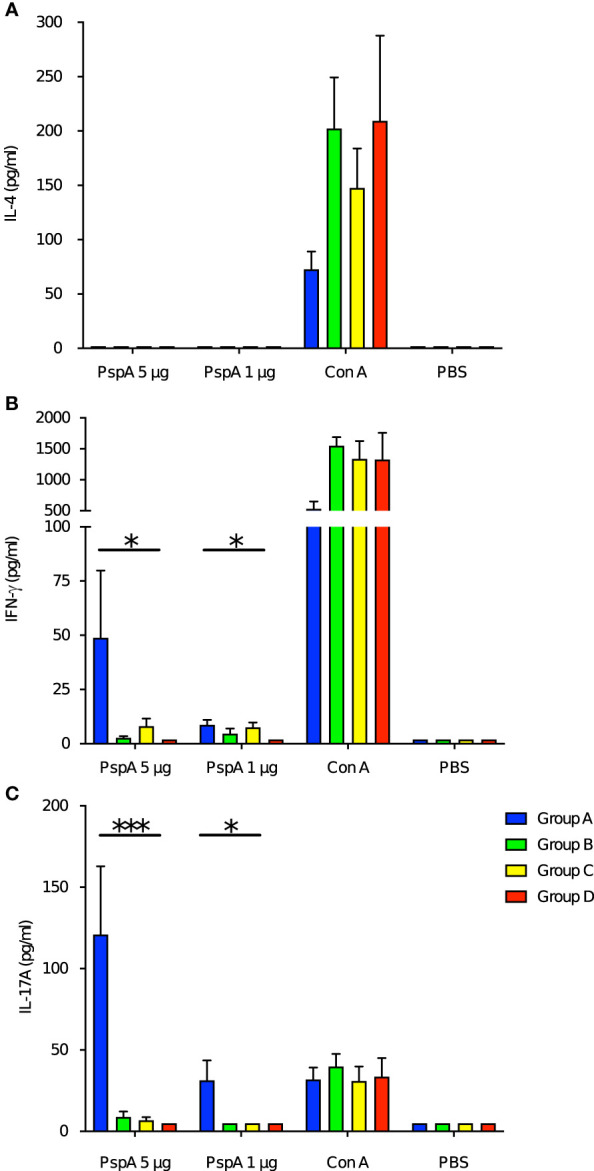
Cytokine production from splenocytes of offspring by PspA stimulation. At the age of week-old, the splenocytes from offspring were incubated with rPspA (1 or 5 µg) or concanavalin A for positive control. Cytokines in the supernatant were quantified by ELISA. **(A)** IL-4, **(B)** IFN-γ, **(C)** IL-17A. Data is shown as mean with standard error. Blue bar, green bar, yellow bar, and red bar represent Group A (n=6), Group B (n=6), Group C (n=6), and Group D (n=6) respectively. Kruskal-Wallis test with Dunn’s multiple comparison was performed for comparison was performed for comparison between the groups. *; *P*<0.05, ***; *P*<0.001.

Next, we investigated the impact of maternal immunization on the cytokine production of the offspring’s splenocytes by PspA stimulation. Pattern of the cytokine production among the offspring in Group A was similar to that among the immunized adult mice. Splenocytes also did not produce IL-4 among all groups of pups (**Figure 5A**). The splenocytes of the pups in Group A significantly produced both IFN-γ and IL-17A after the stimulation with of 1 µg and 5 µg of rPspA (**Figures 5B, C**). When either the birth mother or foster mother was not immunized, enhance of these cytokines was not significant.

### Production of antibodies by PspA stimulation were protective against pneumococcal systemic infection among the offspring derived from immunized mothers

**Figure 6:**
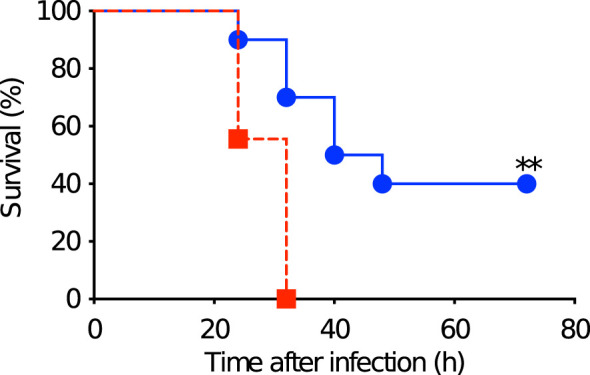
Survival after pneumococcal systemic infection in matured offspring. After two weeks of the subcutaneous stimulation with rPspA (at 6 weeks old), the offspring were intraperitoneally infected with *S. pneumoniae* and monitored every 8 hours until they appeared severely sick. Blue circle with solid line represents offspring derived from and fostered by immunized mother (Group A) (n=10). Red square with dashed line represents offspring derived from and fostered by non-immunized mother (Group D) (n=9). Log rank test was performed for comparison between the two groups. **; *P*<0.01.

The immunity induced by the subcutaneous stimulation with rPspA was investigated in the adult offspring among Group A and D in a pneumococcal systemic infection model. After two weeks of the subcutaneous stimulation with rPspA (at 6 weeks old), the offspring were intraperitoneally infected with *S. pneumoniae* and monitored every 8 hours until they appeared severely sick. All the mice showing severe sick were found bacteremia. The offspring derived from PspA immunized mothers and breastfed by the same mothers survived significantly longer than those from sham-immunized mothers. (*P*=0.0023) (**Figure 6**). On the other hand, all the survived mice in both groups did not have bacteremia at the endpoint of observation.

## Discussion

The induction of immunity to infants by maternal immunization has focused on passive immunity by transferring antibodies from the immunized mother, which temporarily strengthens offspring’s immunity. We previously demonstrated that transferred anti-pneumococcal antibodies from the mother protected pneumococcal colonization and diseases (lung infection and septicemia) during the nursing period (Katsurahara et al., 2008; Hotomi et al., 2011; Kono et al., 2011). In our animal model, IgG played a critical role on the protection against pneumococcal infections. It is consistent that decreased IgG can be a risk of pneumococcal diseases in human. Not only in invasive diseases but also in otitis media, serum IgG is thought to be important as same as secretory IgA (Dhooge et al., 2002; Sharma et al., 2012).

On the other hand, various maternal factors could impact upon immune maturation of offspring (Cabinian et al., 2016; Zambruni et al., 2017; Dawod and Marshall, 2019). These findings prompted a question- do offspring derived from and breastfed by an immunized mother acquire any immune development besides transferred antibodies? Interestingly, offspring derived from immunized mother had the ability to produce anti-PspA specific IgG without direct exposure to pneumococcal antigens. These specific antibody-producing cells are thought to be induced in several ways. This could be by maternal immune cells migrating to offspring transplacentally and/or *via* colostrum; or by maternal chemokines/cytokines promoted immune responses in offspring. Bidirectional migration of maternal/fetal cells between mother and offspring is known as ‘microchimerism’, which is reported as a cause of autoimmune diseases (Gammill and Nelson, 2010; Shrivastava et al., 2019). It was first recognized as a phenomenon when fetal cells from the babies were found in the mothers 27 years after birth. In some cases, cells consisting of male DNA were found in the women and pregnant mothers with systemic sclerosis and polymorphic eruptions (Rak et al., 2009). Microchimeric cells from the mothers have been detected in both healthy and sick fetuses (after the second trimester of pregnancy). In the current study, no significant increase in antibody-producing cells was observed in offspring receiving either transplacental or breastmilk-mediated immunity alone, suggesting that both routes are important for the full functioning of immune cells. On the other hand, a small number of antibody-producing cells were also detected in the control group (Group D). We considered that false positives were detected in ELISpot assay as a result of non-specific immunostimulation because the control group was also administered CTB. Kuipers et al. reported cholera toxin B itself induced immunity against *S. pneumoniae* (Kuipers et al., 2016). As another possibility, antibody producing B cells could be natural B cells that show innately specific response to PspA, or those with a specificity to a similar antigen that can cross-link the B cell receptors.

Ma et al. showed viable maternal immune cells in stomachs of neonates overcame intestinal barriers and finally migrated to the spleen or thymus of offspring, suggesting the possibility of immune maturation of offspring by materno-fetal microchimerism (Ma et al., 2008). Maternal chemokines and cytokines are contained in colostrum together with maternal antibodies and they help maturation of immune organs of offspring (Cabinian et al., 2016; Zambruni et al., 2017; Dawod and Marshall, 2019). Considering non-specific positive cells were also detected in the spleens of offspring derived from sham-immunized mothers (**Figure 2**), general maturation of the immune system by these transferred chemokines and cytokines also affected the current results.

After intranasal immunization, the splenocytes of adult female mice showed enhanced ability to produce cytokines by stimulation with rPspA. Intranasal immunization is known as one of the most effective routes for vaccine because it can induce not only antibody production but also cellular responses such as Th1 and Th17 (Chuang et al., 2018; Habibi et al., 2018). Th17 cells are thought to be important for activating plasmablast and memory B cells after pneumococcal vaccination (Nived et al., 2021). The current results are consistent with these previous reports. Similar characteristics were observed in the splenocytes of offspring derived from immunized mother in terms of the ability of producing antibodies and cytokines without a direct exposure to antigen. Th1 and Th17 play an important role for infant immunity against pneumococcal infections (Basha et al., 2017), and maternal immunization is effective to activate these Th subsets. As we already know intranasal immunization with PspA is able to induce stable immunity in adult mice, this immunization route was adjusted to the current study (Kono et al., 2011). It is not investigated which immunization route is the most effective, however the current study suggested intranasal immunization could induce not only the antibody but also immune memory against PspA among the offspring derived from and fostered by immunized mother.

The offspring still showed higher specific immune responses when they became young adults; this is the first report to suggest maternal immunization could affect immunological memory in their offspring. After a single stimulation of rPspA without any adjuvants, levels of anti-PspA specific antibody in serum continued to increase for two consecutive weeks among the offspring derived from and fostered by immunized mothers that were enough to protect them from lethal challenge by IP infection. This result also shows the effectiveness of boost immunization, even if the offspring’s immune responses were insufficient after termination of breastfeeding.

Maternal immunization has been reported not only favorable points, but also some problems have been pointed out. Among them, a recent study reported that maternal antibody could limit responses of infants to immunization by shaping the early-life B cell repertoire within germinal centers (Vono et al., 2019). Although the offspring derived from and fostered by immunized mother showed a good response in antibody increase and better survival after the pneumococcal challenge, a negative impact of maternal antibodies on infant’s immune maturation should be further investigated.

There are some limitations in this study. The character of anti-PspA specific antibody producing cells in the spleens of seven-day-old offspring could not be confirmed because the number of cells were too low to analyze. It is likely that they are plasmablasts from the time of incubation, but further experiments should be addressed to analyze characteristics of these splenocytes. Another question is ‘Are the splenocytes showing specific responses to PspA from mother or infants?’ Our current results suggest the migration of maternal cells transplacentally or *via* milk, however, the possibility that any fragment of rPspA administered to the mother transmitted to offspring must also be considered. Although it is difficult to exclude this possibility of direct stimulation to offspring by fragments/digestives of rPspA, it is unlikely that protein antigens intranasally administered to female mice at least two weeks before pregnancy would transfer to infants in sufficient number to affect formation of their lasting immunological memory. We used CTB in the current study because it is one of the most representative and basic mucosal adjuvants in animal models. Instead of toxin-based adjuvants, cytokine-based/innate immunity associated adjuvants or delivery vehicles to dendric cells would be good candidates for clinical application. Finally, because a classic method was used to obtain a recombinant protein in the current study, it always contains contamination such as lipopolysaccharide (LPS), which can provoke an immune response *via* toll-like receptor 4 signaling and also directly activate B cells. It is controversial whether any contamination in the purified rPspA may have influenced the results (**Supplementary Figure**). To exclude the possibility that the contamination in the recombinant protein might have enhanced the result of the immunized mother or the offspring derived from and/or fostered by the immunized mother, recent techniques that reduce the endotoxin response in mammalian cells by converting LPS to lipid Iva or a baculovirus expression system should be better strategies for future projects (Ono et al., 2018; Cardoso et al., 2022). Comparing the control group that were sham-immunized with the control extract (from *E. coli* harboring pET20b vector alone) would be another method. Standardization of control group settings in this field using recombinant proteins is an important issue.

Immunological memory is a concept that has been difficult to observe experimentally, but the advancement of science has enabled the understanding of the mechanism, which is based in the antibodies, effector T cells and the priming of B and T cells (Ratajczak et al., 2018). Immune memory starts with the development of CD8+ T cells. Naive T cells expand during the first recognition of the pathogen or antigen and after this pathogen/antigen is cleared from the system, the majority of the effector cells die and memory precursor effector T cells develop and later convert into memory T cells (Pennock et al., 2013). Inherited immune memory would be a milestone in the field of vaccination because the majority of immune cells in infants die quicker than in adults, therefore decreasing the probability of giving rise to the memory T cells (Willems et al., 2009). The current study provided an insight for a novel strategy in the field of maternal immunization with a universal pneumococcal vaccine in order to provide a sustained immunity in offspring.

## Data availability statement

The original contributions presented in the study are included in the article/**Supplementary Material**. Further inquiries can be directed to the corresponding author.

## Ethics statement

The animal study was reviewed and approved by the institutional Animal Care and Use Committee.

## Author contributions

All authors contributed to the article and approved the submitted version. Material preparation and data collection were performed by MK, TI, DM, HS, and DN. MK and MH performed the statistical analysis. The first draft of the manuscript was written by MK and TI. MH commented on the latest version of the manuscript and supervised the study. All authors have read and agreed to the published version of the manuscript.

## Supplementary material

Supplementary Figure 1 Western blotting of serum of immunized mice. rPspA firstly electrophoresed in SDS-PAGE was transferred to a blot paper, then probed with 1:1,000 dilution of the pooled serum obtained from three mice immunized by rPspA. The band at the estimated size of rPspA (*) was confirmed.
