# Supplementary figures and images for: Maternal immunization with pneumococcal surface protein A provides the immune memories of offspring against pneumococcal infection
Source: Front Cell Infect Microbiol. 2023 Mar 23;13:1059603. doi: 10.3389/fcimb.2023.1059603 (PMC10076723; doi:10.3389/fcimb.2023.1059603)

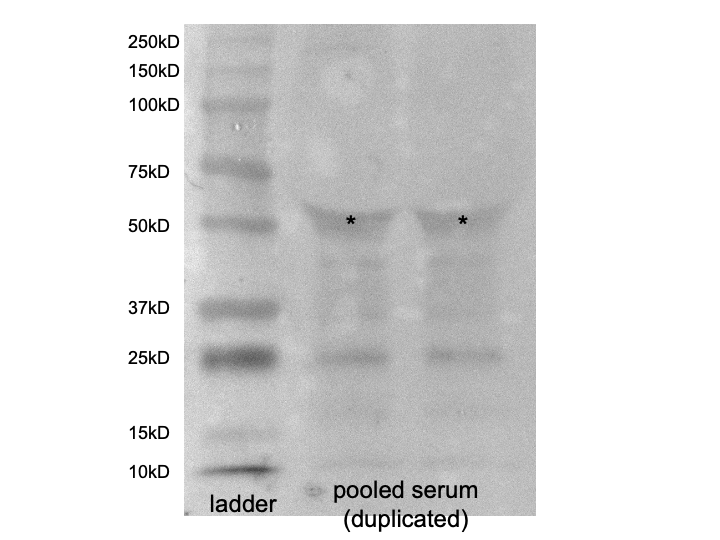

Supplement: Supplementary Figure 1 — Western blotting of serum of immunized mice. rPspA firstly electrophoresed in SDS-PAGE was transferred to a blot paper, then probed with 1:1,000 dilution of the pooled serum obtained from three mice immunized by rPspA. The band at the estimated size of rPspA (*) was confirmed. [file Image_1.tiff]
